# Enumerating Tree-Like Graphs and Polymer Topologies with a Given Cycle Rank

**DOI:** 10.3390/e22111295

**Published:** 2020-11-13

**Authors:** Naveed Ahmed Azam, Aleksandar Shurbevski, Hiroshi Nagamochi

**Affiliations:** Department of Applied Mathematics and Physics, Kyoto University, Kyoto 606-850, Japan; shurbevski@amp.i.kyoto-u.ac.jp (A.S.); nag@amp.i.kyoto-u.ac.jp (H.N.)

**Keywords:** chemical graph, polymer topology, cycle rank, enumeration, canonical representation

## Abstract

Cycle rank is an important notion that is widely used to classify, understand, and discover new chemical compounds. We propose a method to enumerate all non-isomorphic tree-like graphs of a given cycle rank with self-loops and no multiple edges. To achieve this, we develop an algorithm to enumerate all non-isomorphic rooted graphs with the required constraints. The idea of our method is to define a canonical representation of rooted graphs and enumerate all non-isomorphic graphs by generating the canonical representation of rooted graphs. An important feature of our method is that for an integer n≥1, it generates all required graphs with *n* vertices in O(n) time per graph and O(n) space in total, without generating invalid intermediate structures. We performed some experiments to enumerate graphs with a given cycle rank from which it is evident that our method is efficient. As an application of our method, we can generate tree-like polymer topologies of a given cycle rank with self-loops and no multiple edges.

## 1. Introduction

The problem of enumerating discrete structures has several applications in applied fields such as graph theory, chemoinformatics, bioinformatics, and material informatics [1,2,3,4,5,6,7,8,9,10,11]. In particular, the enumeration of chemical compounds is widely used in the discovery of novel drugs [12,13,14,15,16,17] and structural elucidation [18]. It is necessary for an enumeration method to generate all possible required structures without duplication in low computational complexity, due to which designing an enumeration method is not an easy task.

The problem of the enumeration of chemical compounds with given constraints is often modeled as the problem of the enumeration of graphs. Several chemical compound generation methods have been proposed [3,4,5,6,7,8,9], where some methods [3,4] focus on general chemical compounds, while the other methods [5,6,7,8] deal with restricted chemical graphs. These methods are mainly based on the branching algorithm paradigm; the required chemical compounds appear at the leaves of a computation tree. However, these algorithms generate many invalid intermediate structures that appear at the non-leaf nodes of the computation tree [9]. Due to this fact, these methods are inefficient to generate chemical compounds with more than 20 non-hydrogen atoms. Thus, it is natural to explore and develop such methods that can enumerate chemical compounds without generating invalid intermediate structures. Jin et al. [9] proposed one such chemical compound generation method based on the junction tree and the variational autoencoder.

**Figure 1:**
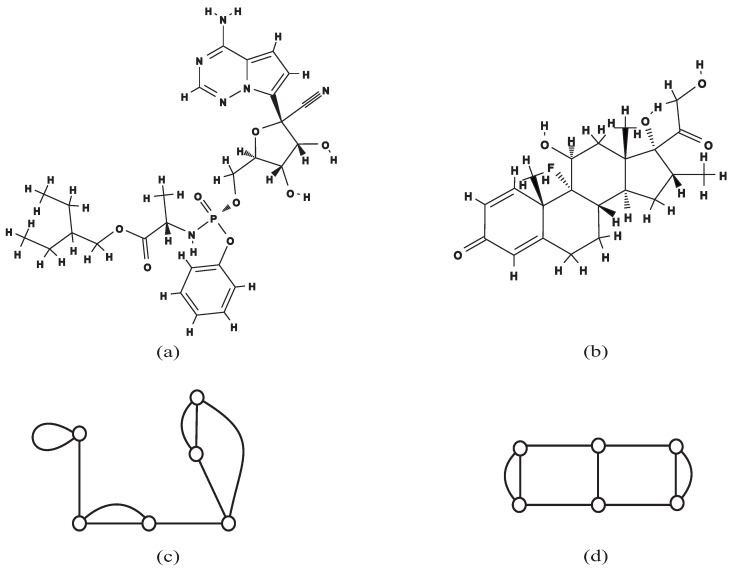
The chemical compounds remdesivir C${}_{27}$H${}_{35}$N${}_{6}$O${}_{8}$P and dexamethasone C${}_{22}$H${}_{29}$FO${}_{5}$ and their polymer topologies: (**a**) the chemical structure of remdesivir C${}_{27}$H${}_{35}$N${}_{6}$O${}_{8}$P taken from PubChem; (**b**) the chemical structure of dexamethasone C${}_{22}$H${}_{29}$FO${}_{5}$ taken from PubChem; (**c**) the polymer topology of remdesivir with cycle rank 4; and (**d**) the polymer topology of dexamethasone with cycle rank 4.

**Figure 2:**
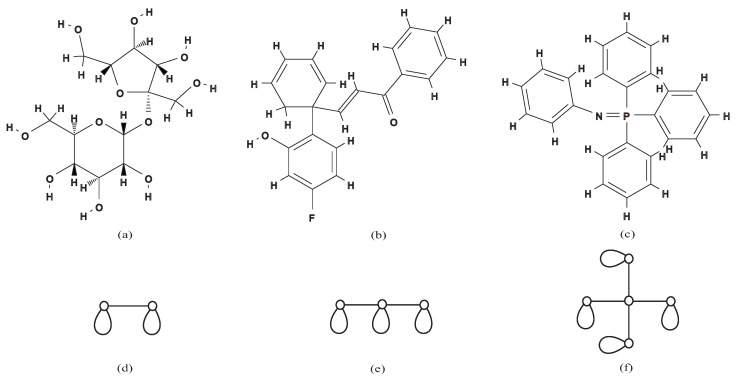
Three chemical compounds with their tree-like polymer topologies containing self-loops: (**a**–**c**) the chemical structures of  C${}_{12}$H${}_{22}$O${}_{11}$, C${}_{21}$H${}_{17}$FO${}_{2}$, and  C${}_{24}$H${}_{20}$NP with CIDs5988, 137,321,354, 75,352, respectively, obtained from the PubChem database; (**d**–**f**) the polymer topologies of the chemical structures in (**a**–**c**) with cycle ranks 2–4, respectively.

For a chemical compound *C*, the *polymer topology* is a connected multi-graph where all vertices have degree at least three obtained by iteratively removing all vertices of degree at most two from *C* [19]. For example, the polymer topologies of the chemical compounds remdesivir C${}_{27}$H${}_{35}$N${}_{6}$O${}_{8}$P (Figure 1a) and dexamethasone C${}_{22}$H${}_{29}$FO${}_{5}$ (Figure 1b) are illustrated in Figure 1c,d, respectively. Observe that two different chemical compounds can have the same polymer topology, and the categorization of polymer topologies can play an important role in understanding and studying the synthetic pathways of macro-chemical compounds [20]. Polymer topologies *P* are often classified with respect to their *cycle rank*, which is the number of edges that are necessary to remove to get a spanning tree of *P*. Haruna et al. [19] developed an enumeration method to generate all polymer topologies with a given rank by using frontier-based search and zero-suppressed decision diagrams. As a result, they enumerated all polymer topologies with cycle rank at most 6. For a multi-graph *G*, we define the *skeleton* to be the simple graph obtained by removing all self-loops and multiple edges from *G*. Notice that the class of graphs with a tree skeleton, Δ≥0 self-loops, and no multiple edges contains all tree-like polymer topologies with cycle rank Δ, and therefore, it is an interesting problem to enumerate all such graphs. Figure 2 illustrates examples of chemical compounds that have tree-like polymer topologies with self-loops and no multiple edges. Recently, Azam et al. [21] proposed a method to count all trees with given numbers of vertices and self-loops by using dynamic programming. As a result, they gave the upper bound and the lower bound on the number of tree-like mutually non-isomorphic polymer topologies with a given rank.

This article aims to develop an efficient method to enumerate all mutually non-isomorphic graphs with a tree skeleton, *n* vertices, Δ self-loops, and no multiple edges without generating invalid intermediate structures. The idea of our method is to define a canonical representation of rooted graphs with the said structures and then enumerate these graphs by generating their canonical representations. As a consequence of our method, we can get all polymer topologies with a tree skeleton, a given cycle rank, self-loops, and no multiple edges. We organize the paper as follows: In Section 2, we discuss some preliminaries. In Section 3, we first prove the mathematical properties based on which we develop our enumeration method. We discuss experimental results and an application of our enumeration method to generate all polymer topologies with a tree skeleton and a given cycle rank in Section 4. We conclude and discuss some future directions in Section 5.

## 2. Preliminaries

For a graph *G*, let V(G) denote the vertex set and E(G) denote the edge set. Let n(G) denote |V(G)| and self(G) denote the number of self-loops in *G*. We define size s(G) of graph *G* to be the sequence (n(G),self(G)). Let s(v) denote the number of self-loops on v∈V(G). For v∈V(G), we denote by $N_{G}\left(v\right)$ the set of neighbors of *v* other than *v*. The *degree* deg${}_{G}\left(v\right)$ of v∈V(G) is defined to be the size of $N_{G}\left(v\right)$.

For a multi-graph *G*, we define the skeleton γ(G) of *G* to be the simple graph obtained by removing all self-loops and multiple edges from the graph *G*. For a rooted graph *G* with root $r_{G}$, we define the *rooted skeleton* γ(G) of *G* to be the rooted simple graph obtained by removing all self-loops from *G* with root $r_{G}$.

Let n≥1 and Δ≥0 be two integers. We denote by H(n,Δ) a maximal set of mutually non-isomorphic rooted graphs with a tree skeleton, *n* vertices, and Δ self-loops. Let *H* be a rooted graph in H(n,Δ). For a vertex v∈V(H), let $H_{v}$ denote the subgraph of *H* rooted at *v* induced by *v* and its descendants in the rooted skeleton γ(H). For a vertex $v\in N_{H}\left(r_{H}\right)$ of root $r_{H}$ of *H*, we call the subgraph $H_{v}$ a *root-subgraph* of *H*.

Let n≥1 and Δ≥0 be two integers and *H* be a rooted graph in H(n,Δ). An *ordered graph* (H,π) of *H* is defined to be the rooted graph *H* with a left-to-right ordering π on the children of each vertex of the rooted skeleton γ(H). Let K=(H,π) be an ordered graph of *H*. For a vertex v∈V(K), we define the ordered subgraph $K_{v}$ of *K* to be a subgraph of *K* rooted at *v* induced by *v* and its descendants in the rooted skeleton γ(K) with preserving the ordering π on the children of each vertex in $\gamma (K_{v})$. For a vertex $v\in N_{K}\left(r_{K}\right)$, we call the ordered subgraph $K_{v}$ an ordered root-subgraph of *K*.

For an ordered tree, we discuss two vertex orderings: depth first search (DFS) ordering [22] and sibling-depth first search (SDFS) ordering. In DFS ordering, we index the vertices of a given ordered tree starting from the root and visiting them from left to right. Masui et al. [23] introduced the SDFS ordering for simple ordered trees. For an ordered tree T=(L,π) with *n* vertices and a left-to-right ordering π, the SDFS ordering is defined to be a vertex ordering obtained by indexing the vertices from the set {1,2,…,n} such that:

(i) the root has index one;
(ii) all siblings are indexed consecutively according to the left-to-right ordering π; and
(iii) all descendants of a vertex *v* are indexed consecutively with indices larger than that of *v* and smaller than the indices of the descendants of any vertex *u*, which is not a descendant of *v* with index larger than *v*.

**Figure 3:**
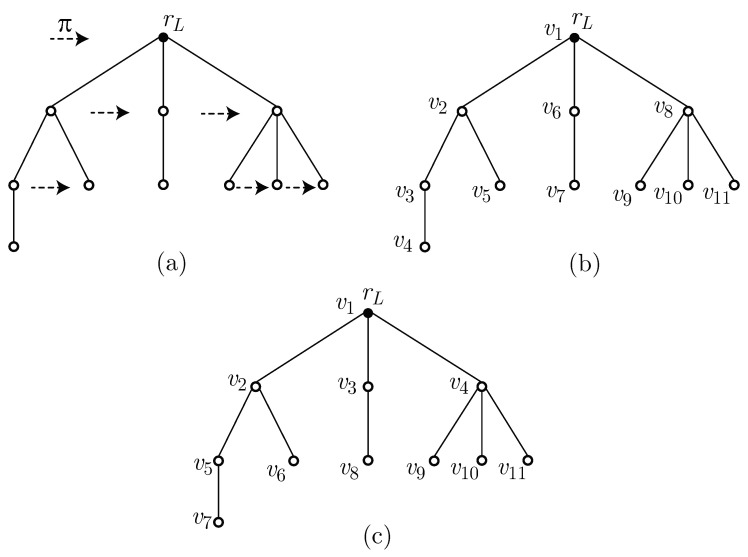
Examples of an ordered tree and vertex indexing: (**a**) an ordered tree T=(L,π) with left-to-right ordering π indicated by the dashed arrow; (**b**) ordered tree T=(L,π) from (**a**) with vertices indexed in depth first search (DFS) order; and (**c**) ordered tree T=(L,π) from (**a**) with vertices indexed in sibling-depth first search (SDFS) order.

Examples of an ordered tree and its vertex indexing in DFS and SDFS ordering are illustrated in Figure 3.

Let $A=(a_{1},a_{2},\ldots ,a_{n})$ and $B=(b_{1},b_{2},\ldots ,b_{m})$ be two sequences over integers. We say that the sequence *A* is lexicographically smaller A≺B than the sequence *B* if there exists an integer *ℓ*, 1≤ℓ≤min{n,m}, such that for each integer *i*, 1≤i≤ℓ, it holds that $a_{i}=b_{i}$ and:

(i) either ℓ=n with n<m or
(ii) ℓ<min{n,m} with $a_{\ell +1}<b_{\ell +1}$.

In such a case, we say that the sequence *B* is lexicographically greater B≻A than the sequence *A*. We define the concatenation A⊕B of the sequences *A* and *B* to be the sequence $\left(a_{1},\ldots ,a_{n},b_{1},\ldots ,b_{m}\right)$.

## 3. Enumeration of Graphs with a Tree Skeleton and a Given Number of Vertices and Self-Loops

For two integers n≥1 and Δ≥0, the aim of this section is to present a method to generate all rooted graphs in H(n,Δ). The idea of our enumeration method is to generate a rooted graph H∈H(n,Δ) by generating a canonical ordered graph of *H*. To achieve this, we define a canonical graph of a rooted graph H∈H(n,Δ) and represent the canonical ordered graph with a sequence by using its ordered subgraphs. Finally, generate the canonical ordered graph of a rooted graph by using the sequence representation of the canonical ordered graph.

We next present a canonical representation of rooted graphs in H(n,Δ) based on a generalization of the canonical representation of simple rooted trees with *n* vertices introduced by Masui et al. [23]. Recall that H(n,0) denote a maximal set of all mutually rooted non-isomorphic simple rooted trees with *n* vertices. Further, note that for Δ≥1, it is necessary for a canonical representation of a rooted graph H∈H(n,Δ) to contain the information of vertices and self-loops in *H*.

Let *H* be a rooted graph in H(n,Δ) and $r_{H}$ denote its root. Further, let K=(H,π) be an ordered graph of *H* with a left-to-right ordering π. For an integer i∈[1,n] and *i*-th vertex $v_{i}$ of *K* following the SDFS ordering on the rooted skeleton γ(K), let $K_{\left(i\right)}$ denote the ordered subgraph $K_{v_{i}}$ of *K* for convenience.

We introduce a canonical representation of *K* by using the information of the number of vertices and self-loops in the ordered subgraphs of *K*. For the vertices $\left\{v_{1},v_{2},\ldots ,v_{n}\right\}$ of *K* indexed by SDFS ordering on the rooted skeleton γ(K), we define the sequence representation SR(*K*) of *K* to be a sequence of the size of each ordered subgraph $K_{\left(i\right)}$, integer i∈[2,n], of *K*:

$$\mathrm{SR}\left(K\right)≜(s\left(K_{\left(2\right)}\right),s\left(K_{\left(3\right)}\right),\ldots ,s\left(K_{\left(n\right)}\right)).$$

**Figure 4:**
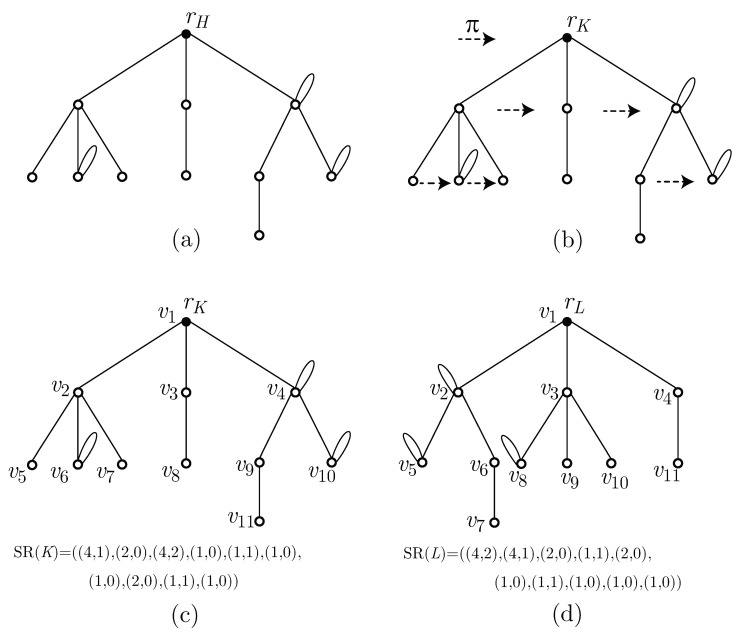
(**a**) A rooted graph H∈H(11,3); (**b**) an ordered graph *K* of *H* with left-to-right ordering π on siblings; (**c**) the ordered graph *K* of *H* with SDFS vertex indexing and SR(K); and (**d**) the canonical representation of *H* and the ordered graph with sequence representation equal to the canonical representation of *H*.

Examples of a rooted graph H∈H(11,3), an ordered graph K=(H,π) of *H* with a left-to-right ordering π, and vertices indexed in SDFS ordering and canonical representation SR(K) of *K* are illustrated in Figure 4a–c.

The next lemma states that the sequence representation of an ordered graph *K* is a concatenation of:

(i) a sequence of the size of the root-subgraphs of *K* in the left-to-right ordering and
(ii) the sequence representation of all root-subgraphs of *K* following the left-to-right ordering.

**Lemma** **1.** *Let K be an ordered graph with n≥1 vertices and Δ≥0 self-loops. For integers $d=\deg_{K}\left(r_{K}\right)$ and i∈[1,d], let $K_{i}$ denote the i-th root-subgraph of K in the left-to-right ordering. Then, it holds that:*

$$\mathrm{SR}\left(K\right)=\left(s\left(K_{1}\right),\ldots ,s\left(K_{d}\right)\right)⊕\mathrm{SR}\left(K_{1}\right)⊕\cdots ⊕\mathrm{SR}\left(K_{d}\right).$$

**Proof.**  We know that in SDFS ordering, the root vertex is indexed by one, and the siblings of a vertex are indexed consecutively. This implies that the subsequence of the first *d* entries of SR(K) is equal to $\left(s\left(K_{1}\right),\ldots ,s\left(K_{d}\right)\right)$. Furthermore, for an integer i=2 (resp., i∈[3,d+1]), the SDFS ordering assigns the index to the descendants of the *i*-th vertex consecutively and greater than the indices of the children (resp., descendants) of the (i−1)-th vertex. From this, it follows that for an integer i=2 (resp., i∈[3,d+1]), the entries of SR$\left(K_{\left(i\right)}\right)$ appear consecutively after the entries of the sequence $s\left(K_{1}\right),\ldots ,s\left(K_{d}\right)$ (resp., SR$\left(K_{\left(i-1\right)}\right)$) in SR(K). This implies that for an integer i∈[1,d], the subsequence of SR(K) consisting of n$(K_{i})-1$ consecutive entries starting from $d+\sum_{1\leq h\leq i-1}n\left(K_{h}\right)-\left(i-1\right)+1$ is actually SR$\left(K_{i}\right)$. Hence, it follows that $\mathrm{SR}\left(K\right)=\left(s\left(K_{1}\right),\ldots ,s\left(K_{d}\right)\right)⊕\mathrm{SR}\left(K_{1}\right)⊕\cdots ⊕\mathrm{SR}\left(K_{d}\right)$. □

For the ordered graph *K* in Figure 4c, SR(K)=((4,1),(2,0),(4,2),(1,0),(1,1),(1,0), (1,0),(2,0),(1,1),(1,0)) and deg${}_{K}\left(r_{K}\right)=3$. This implies that *K* has three root-subgraphs $K_{1}$, $K_{2}$m and $K_{3}$ following the left-to-right ordering. From Figure 4c, we have $\left(s\left(K_{1}\right),s\left(K_{2}\right),s\left(K_{3}\right)\right)=\left(\left(4,1\right),\left(2,0\right),\left(4,2\right)\right)$, SR$\left(K_{1}\right)=\left(\left(1,0\right),\left(1,1\right),\left(1,0\right)\right)$, SR$\left(K_{2}\right)=\left(\left(1,0\right)\right)$, and SR$\left(K_{3}\right)=\left(\left(2,0\right),\left(1,1\right),\left(1,0\right)\right)$. Thus, we see that SR$\left(K\right)=\left(s\left(K_{1}\right),s\left(K_{2}\right),s\left(K_{3}\right)\right)⊕\mathrm{SR}\left(K_{1}\right)⊕\mathrm{SR}\left(K_{2}\right)⊕\mathrm{SR}\left(K_{3}\right)$.

We rephrase the recursion in Lemma 1 for a sequence of pairs in the following paragraph and claim that this recursion is a sufficient condition for a sequence of pairs to be the sequence representation of some ordered graph. We prove this claim in Theorem 1.

Let n≥1 and Δ≥0 be two integers and  $M=(\left(a_{1},b_{1}\right),\left(a_{2},b_{2}\right),\ldots ,\left(a_{n-1},b_{n-1}\right))$ be a sequence of pairs of integers with $a_{i}\geq 1$ and $b_{i}\geq 0$, integer i∈[1,n−2]. We say that the sequence *M* is (n,Δ)-admissible if either n=1 or:

(i) there exists an integer d∈[1,n−1] such that $n-1=\sum_{1\leq i\leq d}a_{i}$ with $\Delta \geq \sum_{1\leq i\leq d}b_{i}$ and
(ii) for each integer i∈[1,d], the subsequence of *M* consisting of $a_{i}-1$ consecutive entries starting from $d+\sum_{1\leq h\leq i-1}a_{h}-\left(i-1\right)+1$ is $\left(a_{i},b_{i}\right)$-admissible.

**Theorem** **1.** *Let n≥1 and Δ≥0 be two integers and $M=(\left(a_{1},b_{1}\right),\left(a_{2},b_{2}\right),\ldots ,\left(a_{n-1},b_{n-1}\right))$ be a sequence of pairs of integers with $a_{i}\geq 1$ and $b_{i}\geq 0$, integer i∈[1,n−2].*

(i) *Sequence M is the sequence representation* SR*(K) of some ordered graph K with n vertices and* Δ *self-loops if and only if M is (n,Δ)-admissible.*
(ii) *Whether M is admissible or not can be tested in O(n) time.*
(iii) *When M is (n,Δ)-admissible, the  ordered graph K with* SR*(K)=M can be constructed in O(n) time.*

**Proof.**  For sequence *M* with an integer d∈[1,n−1] such that $n-1=\sum_{1\leq i\leq d}a_{i}$ and $\Delta \geq \sum_{1\leq i\leq d}b_{i}$, let $M_{i}$ denote the subsequence of *M* consisting of $a_{i}-1$ consecutive entries starting from $d+\sum_{1\leq h\leq i-1}a_{h}-\left(i-1\right)+1$. For an ordered graph *K* and integer $i\in [1,\deg_{K}\left(r_{K}\right)]$, let $K_{i}$ denote the *i*-th root-subgraph of *K* in the left-to-right ordering.

(i) The if-part: Suppose that M=SR(K) for some ordered graph *K* with *n* vertices and Δ self-loops. If n=1, then *M* is (1,Δ)-admissible by the definition of admissibility. Let us assume that n≥2. Then, for $d=\deg_{K}\left(r_{K}\right)$, it holds that $n-1=\sum_{1\leq i\leq d}n\left(K_{i}\right)$ and $\Delta \geq \sum_{1\leq i\leq d}s\left(K_{i}\right)$. Further, by Lemma 1 for each integer i∈[1,d], the subsequence $M_{i}$ of *M* is equal to SR$\left(K_{i}\right)$. Thus, by recursively using Lemma 1 for SR$\left(K_{i}\right)$, i∈[1,d], we see that the sequence representation $\mathrm{SR}(K_{i})$ is $\left(n\left(K_{i}\right),s\left(K_{i}\right)\right)$-admissible. Hence, it follows that the sequence representation SR(K) is (n,Δ)-admissible.

The only-if part: We prove the converse of (i) by induction on *n*.

For n=1, *M* is (1,Δ)-admissible by the definition of admissibility. Note that *M* is an empty sequence in this case. Let *K* be an ordered graph with n=1 vertices and Δ self-loops. Then, SR(K) is an empty sequence, and hence, M=SR(K).

Suppose that the converse of (i) holds for any positive integer *ℓ*. We show that the converse holds for the integer ℓ+1. Let *M* be (ℓ+1,Δ)-admissible. Then, by the definition of admissibility, there exists an integer d∈[1,ℓ] such that $\ell =\sum_{1\leq i\leq d}a_{i}$ and $\Delta \geq \sum_{1\leq i\leq d}b_{i}$. This implies that for an integer i∈[1,d], we have $a_{i}\leq \ell$. Further, for each integer i∈[1,d], the subsequence $M_{i}$ is $\left(a_{i},b_{i}\right)$-admissible by the admissibility of *M*. This and the inductive hypothesis that the converse of (i) holds for any integer ℓ≥1 imply that for each integer i∈[1,d], there exists an ordered graph *H* with $a_{i}$ vertices and $b_{i}$ self-loops such that $M_{i}=\mathrm{SR}\left(H\right)$. Let *K* denote the ordered graph with ℓ+1 vertices, Δ self-loops, deg${}_{K}\left(r_{K}\right)=d$, $\Delta -\sum_{1\leq i\leq d}b_{i}$ self-loops on the root $r_{K}$, and the  *i*-th root subgraph $K_{i}$ of *K* be the ordered subgraph *H* such that $M_{i}=\mathrm{SR}\left(H\right)$. Then, it immediately follows that:

$$\begin{array}{c} \mathrm{SR}\left(K\right)=\left(s\left(K_{1}\right),\ldots ,s\left(K_{d}\right)\right)⊕M_{1}⊕M_{2}⊕\ldots ⊕M_{d}. \end{array}$$

This means that M=SR(K) holds, since $\left(\left(a_{1},b_{1}\right),\ldots ,\left(a_{d},b_{d}\right)\right)=\left(s\left(K_{1}\right),\ldots ,s\left(K_{d}\right)\right)$, showing that the converse holds for the integer ℓ+1.

Hence, by mathematical induction, the converse of (i) holds for any integer n≥1.

(ii)  We prove this result by induction on *n*.

For n=1, the sequence *M* is an empty sequence and is (1,Δ)-admissible by the definition of admissibility. Therefore, it takes constant O(1) time to test admissibility in this case.

Suppose that for n=ℓ, ℓ≥1, the admissibility of sequence *M* can be tested in O(ℓ) time. We show that the statement (ii) holds for n=ℓ+1. To show if *M* is (ℓ+1,Δ)-admissible, we need to find an integer d∈[1,ℓ] such that $\ell =\sum_{1\leq i\leq d}a_{i}$ and $\Delta \geq \sum_{1\leq i\leq d}b_{i}$. Such an integer *d* can be identified in O(d) time. Suppose that such an integer *d* exists for *M*. Then, for each integer i∈[1,d], we next need to test if the subsequence $M_{i}$ is $\left(a_{i},b_{i}\right)$-admissible. Note that the size of the sequence $M_{i}$ is $a_{i}-1$, i∈[1,d]. By $\ell =\sum_{1\leq i\leq d}a_{i}$, it holds that $a_{i}\leq \ell$, i∈[1,d]. This and the inductive hypothesis imply that for an integer i∈[1,d], the admissibility of the sequence $M_{i}$ can be tested in $O(a_{i})$ time. Thus, the time testing admissibility of *M* is $O\left(d+\sum_{1\leq i\leq d}a_{i}\right)=O\left(d+\ell \right)=O\left(\ell +1\right)$, since d≤ℓ.

Hence, by mathematical induction, the admissibility of a sequence *M* of size n−1, n≥1 can be tested in O(n) time.

(iii) We prove the claim in (iii) by induction on *n*.

For n=1, *M* is (1,Δ)-admissible and is the sequence representation of the ordered graph *K* with only one vertex and Δ self-loops. This implies that *K* can be constructed in O(1) time.

Suppose that for n=ℓ, ℓ≥1, the  statement (iii) holds. We show that the statement (iii) holds for n=ℓ+1. Let *M* be an (ℓ+1,Δ)-admissible sequence. Then, there exists an integer d∈[1,ℓ], such that $\ell =\sum_{1\leq i\leq d}a_{i}$ and $\Delta \geq \sum_{1\leq i\leq d}b_{i}$. By (i), there exists an ordered graph *K* with *n* vertices and Δ self-loops such that SR(K)=M. Thus, it holds that $\deg_{K}\left(r_{K}\right)=d$. Further, by Lemma 1, for each integer i∈[1,d], the *i*-th root-subgraph $K_{i}$ of *K* has $a_{i}$ vertices and $b_{i}$ self-loops. Observe that such an integer *d* exists uniquely due to the admissibility of *M*. This implies that $\deg_{K}\left(r_{K}\right)$ can be obtained in O(d) time.

By SR(K)=M and Lemma 1, for each integer i∈[1,d], it holds that SR$\left(K_{i}\right)=M_{i}$. Recall that the size of SR$\left(K_{i}\right)$ is n$(K_{i})-1$, which is equal to $a_{i}-1$, i∈[1,d]. Further, by $\ell =\sum_{1\leq i\leq d}a_{i}$, it follows that $a_{i}\leq \ell$, i∈[1,d]. Thus, by the inductive hypothesis for an integer i∈[1,d], the subgraph $K_{i}$ can be constructed from $M_{i}$ in $O(a_{i})$ time. Since $d+\sum_{1\leq i\leq d}a_{i}=d+\ell$ and d≤ℓ, *K* can be constructed in O(ℓ+1) time from *M*.

Hence, by mathematical induction, for integers n≥1 and Δ≥0 and an (n,Δ)-admissible sequence *M*, the ordered graph *K* with SR(K)=M can be constructed by *M* in O(n) time. □

Let $M=(\left(a_{1},b_{1}\right),\left(a_{2},b_{2}\right),\ldots ,\left(a_{n-1},b_{n-1}\right))$ be an (n,Δ)-admissible sequence. Then, there exists an integer d∈[1,n−1] such that $n-1=\sum_{1\leq i\leq d}a_{i}$. Furthermore, by Theorem 1(i), there exists an ordered graph *K* with *n* vertices and Δ self-loops such that deg${}_{K}\left(r_{K}\right)=d$ and SR(K)=M. Note that such an integer *d* and ordered graph *K* are unique. We call the integer *d* the root-degree of *M* and denote it by d(M). Moreover, for each integer i∈[1,d(M)], the subsequence of *M* consisting of $a_{i}-1$ consecutive entries starting from $d\left(M\right)+\sum_{1\leq h\leq i-1}a_{h}-\left(i-1\right)+1$ is equal to the sequence representation of the *i*-th root-subgraph of *M*. For an integer i∈[1,d(M)], we call such a subsequence of *M* the *i*-th root-subsequence of *M* and denote it by M(i).

By Theorem 1(i), it follows that an ordered graph *K* with n≥1 vertices and Δ≥0 self-loops can be completely determined by SR(K). Thus, we define a canonical representation of a rooted graph H∈H(n,Δ) as follows. For a rooted graph H∈H(n,Δ), we define the canonical representation to be an (n,Δ)-admissible sequence *M* such that *M* is lexicographically maximum among all (n,Δ)-admissible sequences that are the sequence representation of ordered graphs of *H*.

In Figure 4d, we show the canonical representation *M* of the rooted graph H∈H(11,3) illustrated in Figure 4a. Further, we show the ordered graph *L* such that SR(L)=M.

To generate all rooted graphs in H(n,Δ), it is enough to generate the canonical representation of each rooted graph H∈H(n,Δ) by Theorem 1(i). For two integers n≥1 and Δ≥0, let M(n,Δ) denote the set of all (n,Δ)-admissible sequences that are canonical representation of graphs in H(n,Δ). Note that the empty sequence is the only sequence in M(1,Δ). In the next lemma, we give a characterization of sequences in M(n,Δ).

**Lemma** **2.** *Let n≥2 and Δ≥0 be two integers. Let $M=(\left(a_{1},b_{1}\right),\left(a_{2},b_{2}\right),\ldots ,\left(a_{n-1},b_{n-1}\right))$ be a sequence of integer pairs with an integer d∈[1,n−1] such that $n-1=\sum_{1\leq i\leq d}a_{i}$. For an integer i∈[1,d], let M(i) denote the subsequence of M consisting of $a_{i}-1$ consecutive entries starting from $d+\sum_{1\leq h\leq i-1}a_{h}-\left(i-1\right)+1$. Then, M∈M(n,Δ) if and only if the following hold:*

(i) *$a_{i}\geq 1,\forall i\in \left[1,d\right]$, $\sum_{1\leq i\leq d}a_{i}=n-1$ and $a_{i}\geq a_{i+1},\forall i\in \left[1,d-1\right]$;*
(ii) *$b_{i}\geq 0,\forall i\in \left[1,d\right]$, $\sum_{1\leq i\leq d}b_{i}\leq \Delta$ and for each integer i∈[1,d−1] such that $a_{i}=a_{i+1}$, it holds that $b_{i}\geq b_{i+1}$; and*
(iii) *$M\left(i\right)\in M\left(a_{i},b_{i}\right),\forall i\in \left[1,d\right]$, and for each integer i∈[1,d−1] such that $a_{i}=a_{i+1}$ and $b_{i}=b_{i+1}$, it holds that M(i)⪰M(i+1).*

**Proof.**  The if part: Let M∈M(n,Δ). Then, by the definition of admissibility, it holds that d=d(M). Let *H* denote the ordered graph with *n* vertices and Δ self-loops such that SR(H)=M.

(i) By the admissibility of *M*, we have $a_{i}\geq 1,\forall i\in \left[1,d\left(M\right)\right]$, $\sum_{1\leq i\leq d(M)}a_{i}=n-1$. Furthermore, *M* is the canonical representation of *H*, and therefore, for the sequence representation $\left(\left(s_{1},s_{1}^{\prime }\right),\left(s_{2},s_{2}^{\prime }\right),\ldots ,\left(s_{n-1},s_{n-1}^{\prime }\right)\right)$ of any ordered graph of *H*, it holds that $\left(a_{1},\ldots ,a_{d(M)}\right)⪰\left(s_{1},\ldots ,s_{d(M)}\right)$. This eventually implies that $a_{i}\geq a_{i+1},\forall i\in \left[1,d-1\right]$.
(ii) By the admissibility of *M*, it holds that $b_{i}\geq 0,\forall i\in \left[1,d\left(M\right)\right]$, $\sum_{1\leq i\leq d(M)}b_{i}\leq \Delta$. Moreover, for the sequence representation $\left(\left(s_{1},s_{1}^{\prime }\right),\left(s_{2},s_{2}^{\prime }\right),\ldots ,\left(s_{n-1},s_{n-1}^{\prime }\right)\right)$ of any ordered graph of *H* such that $s_{i}=a_{i},\forall i\in \left[1,d\left(M\right)\right]$, it holds that $\left(b_{1},\ldots ,b_{d(M)}\right)⪰\left(s_{1}^{\prime },\ldots ,s_{d(M)}^{\prime }\right)$ since *M* is the canonical representation of *H*. This implies that for each integer i∈[1,d(M)−1] such that $a_{i}=a_{i+1}$, it holds that $b_{i}\geq b_{i+1}$.
(iii) We first prove that for each integer i∈[1,d(M)], it holds that $M\left(i\right)\in M\left(a_{i},b_{i}\right)$.

For an integer i∈[1,d(M)], let $H_{i}$ denote the *i*-th root-subgraph of *H* following the left-to-right ordering. Then, by Lemma 1 for each integer i∈[1,d(M)], it holds that $\mathrm{SR}\left(H_{i}\right)=M\left(i\right)$.

Suppose on the contrary that there exists an integer i∈[1,d(M)] such $M\left(i\right)\notin M\left(a_{i},b_{i}\right)$. This means that there exists an ordered graph *L* that is rooted isomorphic to $H_{i}$, and SR(L)≻M(i) holds. Let $M^{\prime }$ denote the sequence obtained from *M* by replacing the subsequence $M_{i}$ with SR(L). Clearly, $M^{\prime }$ is (n,Δ)-admissible, and $M^{\prime }≻M$ holds by the construction of $M^{\prime }$. Let $H^{\prime }$ denote the ordered graph obtained by replacing $H_{i}$ with *L* in *H* and preserving the ordering of the children of each vertex in *L*. Then, we see that $H^{\prime }$ is rooted isomorphic to *H*, and SR$\left(H^{\prime }\right)=M^{\prime }$. This contradicts the fact that *M* is a sequence in M(n,Δ). Hence, for each integer i∈[1,d(M)], it holds that $M\left(i\right)\in M\left(a_{i},b_{i}\right)$.

Recall that *M* is the canonical representation of *H*. This implies that for the sequence representation $\left(\left(s_{1},s_{1}^{\prime }\right),\left(s_{2},s_{2}^{\prime }\right),\ldots ,\left(s_{n-1},s_{n-1}^{\prime }\right)\right)$ of any ordered graph of *H* such that $\left(s_{i},s_{i}^{\prime }\right)=\left(a_{i},b_{i}\right),\forall i\in \left[1,d\left(M\right)\right]$, it holds that $\left(\left(a_{d(M)+1},b_{d(M)+1}\right),\ldots ,\left(a_{n-1},b_{n-1}\right)\right)⪰\left(\left(s_{d(M)+1},s_{d(M)+1}^{\prime }\right),\ldots ,\left(s_{n-1},s_{n-1}^{\prime }\right)\right)$. This implies that for each integer i∈[1,d(M)−1] such that $a_{i}=a_{i+1}$ and $b_{i}=b_{i+1}$, we have M(i)⪰M(i+1).

The only-if part: Let *M* satisfy (i), (ii), and (iii). We show that M∈M(n,Δ). To prove this, we show that *M* is a canonical representation of some graph in H(n,Δ).

By (i) and (ii), we have $a_{i}\geq 1,b_{i}\geq 0,\forall i\in \left[1,d\right]$, $n-1=\sum_{1\leq i\leq d}a_{i}$ and $\Delta \geq \sum_{1\leq i\leq d}b_{i}$. Furthermore, for each integer i∈[1,d], the sequence $M_{i}$ is $\left(a_{i},b_{i}\right)$-admissible, since $M\left(i\right)\in M\left(a_{i},b_{i}\right)$ by (iii). This implies that *M* is (n,Δ)-admissible.

By Theorem 1(i), there exists a unique ordered graph K=(H,π) such that SR(K)=M for some H∈H(n,Δ). This implies that deg${}_{K}\left(r_{K}\right)=d$, and for each integer i∈[1,d], the *i*-th root subgraph of *K* has $a_{i}$ vertices and $b_{i}$ self-loops. This implies that any ordered graph *L* that is rooted isomorphic to *K* has *x* vertices and *y* self-loops such that $\left(x,y\right)=\left(a_{i},b_{i}\right)$ for some i∈[1,d].

The condition $a_{i}\geq a_{i+1},\forall i\in \left[1,d-1\right]$ in (i) implies that for the sequence representation $S=(\left(s_{1},s_{1}^{\prime }\right),\left(s_{2},s_{2}^{\prime }\right),\ldots ,\left(s_{n-1},s_{n-1}^{\prime }\right))$ of any ordered graph that is rooted isomorphic to *K*, it holds that M⪰S since $\left(a_{1},\ldots ,a_{d}\right)⪰\left(s_{1},\ldots ,s_{d}\right)$. For the condition for each integer i∈[1,d−1] such that $a_{i}=a_{i+1}$, it holds that $b_{i}\geq b_{i+1}$ in (ii) implies that for the sequence representation $S=(\left(s_{1},s_{1}^{\prime }\right),\left(s_{2},s_{2}^{\prime }\right),\ldots ,\left(s_{n-1},s_{n-1}^{\prime }\right))$ of any ordered graph that is rooted isomorphic to *K* such that $s_{i}=a_{i},\forall i\in \left[1,d\right]$, it holds that M⪰S since $\left(b_{1},\ldots ,b_{d}\right)⪰\left(s_{1}^{\prime },\ldots ,s_{d}^{\prime }\right)$. Finally, for the condition for each integer i∈[1,d−1] such that $a_{i}=a_{i+1}$ and $b_{i}=b_{i+1}$, it holds that M(i)⪰M(i+1) in (iii) implies that for the sequence representation $S=(\left(s_{1},s_{1}^{\prime }\right),\left(s_{2},s_{2}^{\prime }\right),\ldots ,\left(s_{n-1},s_{n-1}^{\prime }\right))$ of any ordered graph that is rooted isomorphic to *K* and $\left(s_{i},s_{i}^{\prime }\right)=\left(a_{i},b_{i}\right),\forall i\in \left[1,d\right]$, it holds that M⪰S since $\left(\left(a_{d+1},b_{d+1}\right),\ldots ,\left(a_{n-1},b_{n-1}\right)\right)⪰\left(\left(s_{d+1},s_{d+1}^{\prime }\right),\ldots ,\left(s_{n-1},s_{n-1}^{\prime }\right)\right)$. This eventually implies that *M* is the canonical representation of *H* from which it follows that M∈M(n,Δ). □

We next give the structure of the sequences that are lexicographically minimum and maximum among all sequences in M(n,Δ).

**Lemma** **3.** *Let n≥2 and Δ≥0 be two integers.*

(i) *The sequences N=((1,0),(1,0),…,(1,0)) and M=((n−1,Δ),(n−2,Δ),…,(1,Δ)) each of length n−1 are lexicographically minimum and maximum among all sequences in M(n,Δ), respectively.*
(ii) *Whether a sequence in M(n,Δ) is lexicographically minimum or maximum among all sequences in M(n,Δ) can be tested in O(n).*

**Proof.** 

(i) It is easy to observe that the sequence *N* is (n,Δ)-admissible. Furthermore, for two integers i≥2 and j≥0, the ranges of the first and the second entries in any sequence in M(i,j) are [1,i−1] and [0,j], respectively. This implies that the sequence *N* is lexicographically minimum among all the sequences in M(n,Δ). Moreover, the sequence ((1,Δ)) is admissible and lexicographically maximum among all the sequences in M(2,Δ). From this, it follows that the sequence ((2,Δ),(1,Δ)) is admissible and lexicographically maximum among all the sequences in M(3,Δ). Thus, by using this inductive argument, we can conclude that the sequence *M* is admissible and lexicographically maximum among all the sequences in M(n,Δ).
(ii) We know that a sequence *S* in M(n,Δ) is of length n−1, and therefore, by using a for-loop of size n−1 and (i), we can test if *S* is lexicographically minimum or maximum among all sequences in M(n,Δ) in O(n) time.

 □

Let S(n,Δ) and L(n,Δ) denote the lexicographically minimum and maximum sequences among all sequences in M(n,Δ), respectively.

For a sequence M∈M(n,Δ) such that M≠S(n,Δ), we define the predecessor P(M) of *M* to be the sequence that is lexicographically maximum among all sequences that are lexicographically smaller than *M*, i.e., there does not exist a sequence N∈M(n,Δ)\{P(M)} such that M≻N≻P(M) holds.

For a sequence M∈M(n,Δ), we next give the structure of the predecessor P(M), if it exists, of *M*.

**Theorem** **2.** *Let n≥2 and Δ≥0 be two integers and $M=(\left(a_{1},b_{1}\right),\left(a_{2},b_{2}\right),\ldots ,\left(a_{n-1},b_{n-1}\right))$ be a sequence in M(n,Δ). Let d denote the root-degree of M. Then, for the predecessor* P*$\left(M\right)=(\left(x_{1},y_{1}\right),\left(x_{2},y_{2}\right),\ldots ,\left(x_{n-1},y_{n-1}\right))$, if it exists, we have:*

(a) *If $a_{i}=1$ and $b_{i}=0$, ∀i∈[1,d], then* P*(M) does not exist.*
(b) *If $a_{i}\neq 1$ for some i∈[1,d], $b_{j}=0$ and $M\left(j\right)=S\left(a_{j},b_{j}\right)$, ∀j∈[1,d]. Then, for the largest integer k∈[1,d] such that $a_{k}\neq 1$, it holds that* d(P*$\left(M\right))=k-1+\left\lceil \left(a_{k}+d-k\right)/\left(a_{k}-1\right)\right\rceil$, $y_{1}=\Delta$, $y_{i}=0,\forall i\in \left[2,d(P\left(M\right)\right)]$, $x_{i}=a_{i},\forall i\in \left[1,k-1\right]$, $x_{i}=a_{k}-1,\forall i\in \left[k,d(P\left(M\right)\right)-1]$, $x_{d(P(M))}=a_{k}+d-k-\left\lfloor \left(a_{k}+d-k\right)/\left(a_{k}-1\right)\right\rfloor \left(a_{k}-1\right)$, and* P*$\left(M\right)\left(i\right)=L\left(x_{i},y_{i}\right)$, ∀i∈[1,d(P(M))].*
(c) *If $b_{i}\neq 0$ for some i∈[1,d], $M\left(j\right)=S\left(a_{j},b_{j}\right)$, ∀j∈[1,d]. For the largest integer k∈[1,d] such that $b_{k}\neq 0$, let $p≜\max\{i\leq d|a_{k}=a_{k+i}\}$, q≜p+1 if $b_{k}=1$, and $q≜\lfloor \left(\Delta -\sum_{1\leq i\leq k-1}b_{i}\right)/\left(b_{k}-1\right)\rfloor$ if $b_{k}\geq 2$ and t≜min{q,p+1}. Then, it holds that* d(P*(M))=d, $x_{i}=a_{i},\forall i\in \left[1,d\right]$, $y_{i}=b_{i},\forall i\in \left[1,k-1\right]$, $y_{k}=b_{k}-1$; for k≠d, we have $y_{k+i}=b_{k}-1,\forall i\in \left[1,t-1\right]$, $y_{k+t}=\left(\Delta -\sum_{1\leq i\leq k-1}b_{i}\right)-t\left(b_{k}-1\right)$, $y_{i}=0,\forall i\in \left[k+t+1,d\right]$, and* P*$\left(M\right)\left(i\right)=L\left(x_{i},y_{i}\right)$, ∀i∈[1,d].*
(d) *Otherwise if $a_{i}\neq 1,b_{j}\neq 0$, $M\left(\ell \right)\neq S\left(a_{\ell },b_{\ell }\right)$, for some i,j,ℓ∈[1,d]. For the largest integer k∈[1,d] such that $M\left(k\right)\neq S\left(a_{k},b_{k}\right)$, let $p≜\max\{i\leq d|a_{k}=a_{k+i}\;\mathrm{and}\;b_{k}=b_{k+1}\}$. Then, it holds that* d(P*(M))=d, $\left(x_{i},y_{i}\right)=\left(a_{i},b_{i}\right),\forall i\in \left[1,d\right]$,* P*(M)(i)=M(i), ∀i∈[1,k−1],* P*(M)(k+i)=P(M(k)),∀i∈[0,p], and* P*$\left(M\right)\left(i\right)=L\left(a_{i},b_{i}\right)$, ∀i∈[k+p+1,d].*

**Proof.** 

(a) If $a_{i}=1$ and $b_{i}=0$, ∀i∈[1,d], then it holds that d=n−1. Thus, by Lemma 3(i), it holds that M=S(n,Δ), and therefore, P(M) does not exist.
(b) If $a_{i}\neq 1$ for some i∈[1,d], $b_{j}=0$ and $M\left(j\right)=S\left(a_{j},b_{j}\right)$, ∀j∈[1,d], then *M* is lexicographically minimum among all those sequences $\left(\left(c_{1},c_{1}^{\prime }\right),\ldots ,\left(c_{n-1},c_{n-1}^{\prime }\right)\right)\in M\left(n,\Delta \right)$ for which it holds that $c_{i}=a_{i},\forall i\in \left[1,d\right]$. Further, $a_{i}\neq 1$ for some i∈[1,d] implies that P(M) exists. By the definition of a predecessor, observe that P(M) is lexicographically maximum among all those sequences $S=\left(\left(s_{1},s_{1}^{\prime }\right),\ldots ,\left(s_{n-1},s_{n-1}^{\prime }\right)\right)\in M\left(n,\Delta \right)$ for which it holds that $s_{i}=x_{i},\forall i\in \left[1,d(P\left(M\right)\right)]$. This implies that for each such sequence *S*, it holds that either $\left(y_{1},y_{2},\ldots ,y_{d(P(M))}\right)≻\left(s_{1}^{\prime },s_{2}^{\prime },\ldots ,s_{d(P(M))}^{\prime }\right)$ or $y_{i}=s_{i}^{\prime }$ and P(M)(i)⪰S(i),∀i∈[1,d(P(M))]. The former implies that $y_{1}=\Delta$, $y_{i}=0,\forall i\in \left[2,d(P\left(M\right)\right)]$, while the latter implies that P$\left(M\right)\left(i\right)=L\left(x_{i},y_{i}\right)$, ∀i∈[1,d(P(M))]. Further, the sequence $\left(x_{1},\ldots ,x_{d(P(M))}\right)$ satisfies Lemma 2(i), and there does not exist a sequence *L* that satisfies Lemma 2(i) such that $\left(x_{1},\ldots ,x_{d(P(M))}\right)≺L≺\left(a_{1},\ldots ,a_{d}\right)$ holds by the definition of P(M). This and the definition of *k* imply that $x_{i}=a_{i},\forall i\in \left[1,k-1\right]$; the sequence $\left(x_{k},x_{k+1},\ldots ,x_{d(P(M))}\right)$ is a non-decreasing sequence, and there there does not exist a sequence $Z=(z_{1},\ldots ,z_{t})$ such that $z_{i}\in \left[1,a_{k}-1\right],\forall i\in \left[1,t\right]$ and $\sum_{1\leq i\leq t}z_{i}=\sum_{k\leq j\leq d}a_{j}$ for which it holds that $\left(x_{k},\ldots ,x_{d(P(M))}\right)≺Z≺\left(a_{k},\ldots ,a_{d}\right)$ holds. This eventually implies that d(P$\left(M\right))=k-1+\left\lceil \sum_{k\leq i\leq d}a_{i}/\left(a_{k}-1\right)\right\rceil$, $x_{i}=a_{k}-1,\forall i\in \left[k,d(P\left(M\right)\right)-1]$, and $x_{d(P(M))}=\sum_{k\leq i\leq d}a_{i}-\left\lfloor \sum_{k\leq i\leq d}a_{i}/\left(a_{k}-1\right)\right\rfloor \left(a_{k}-1\right)$. Thus, by the definition of *k*, we have $\sum_{k\leq i\leq d}a_{i}=a_{k}+d-k$, and therefore, we have the required result.
(c) If $b_{i}\neq 0$ for some i∈[1,d] and $M\left(j\right)=S\left(a_{j},b_{j}\right)$, ∀j∈[1,d], then *M* is lexicographically minimum among all those sequences $\left(\left(c_{1},c_{1}^{\prime }\right),\ldots ,\left(c_{n-1},c_{n-1}^{\prime }\right)\right)\in M\left(n,\Delta \right)$ for which it holds that $\left(c_{i},c_{i}^{\prime }\right)=\left(a_{i},b_{i}\right),\forall i\in \left[1,d\right]$. Since $b_{i}\neq 0$ for some i∈[1,d], therefore P(M) exists and is lexicographically maximum among all those sequences $\left(\left(s_{1},s_{1}^{\prime }\right),\ldots ,\left(s_{n-1},s_{n-1}^{\prime }\right)\right)\in M\left(n,\Delta \right)$ for which it holds that $\left(s_{i},s_{i}^{\prime }\right)=\left(a_{i},y_{i}\right),\forall i\in \left[1,d(P\left(M\right)\right)]$. This implies that $x_{i}=a_{i},\forall i\in \left[1,d(P\left(M\right)\right)]$, and therefore, we have d(P(M))=d. Furthermore, $\left(y_{1},y_{2},\ldots ,y_{d}\right)≻\left(s_{1}^{\prime },s_{2}^{\prime },\ldots ,s_{d}^{\prime }\right)$ and P(M)(i)⪰S(i),∀i∈[1,d]. This implies that for each integer i∈[1,d], it holds that P$\left(M\right)\left(i\right)=L\left(a_{i},y_{i}\right)$, and the sequence $\left(y_{1},y_{2},\ldots ,y_{d}\right)$ is lexicographically minimum among all those sequences that satisfy Lemma 2(ii) and are lexicographically smaller than $\left(b_{1},b_{2},\ldots ,b_{d}\right)$. This and the definition of *k* imply that $y_{i}=b_{i},\forall i\in \left[1,k-1\right]$; by Lemma 2(ii), the sequence $\left(y_{k},y_{k+1},\ldots ,y_{k+p}\right)$ is a non-decreasing sequence, and there does not exist a sequence $Z=(z_{k},z_{k+1},\ldots ,z_{k+p})$ such that $z_{i}\in \left[0,b_{k}-1\right]$ and $\sum_{k\leq i\leq k+p}z_{i}=\min\left\{\left(p+1\right)\left(b_{k}-1\right),\Delta -\sum_{1\leq i\leq k-1}b_{i}\right\}$ for which it holds that $\left(y_{k},y_{k+1}\ldots ,x_{k+p}\right)≺Z≺\left(b_{k},b_{k+1},\ldots ,b_{k+p}\right)$. This eventually implies that $y_{k}=b_{k}-1$ and for k≠d $y_{k+i}=b_{k}-1,\forall i\in \left[1,t-1\right]$. This and the minimality of $\left(y_{1},y_{2},\ldots ,y_{d}\right)$ imply that $y_{k+t}=\left(\Delta -\sum_{1\leq i\leq k-1}b_{i}\right)-t\left(b_{k}-1\right)$ and $y_{i}=0,\forall i\in \left[k+t+1,d\right]$.
(d) The conditions of this case imply that *M* is not the lexicographically minimum among all those sequences $\left(\left(c_{1},c_{1}^{\prime }\right),\ldots ,\left(c_{n-1},c_{n-1}^{\prime }\right)\right)\in M\left(n,\Delta \right)$ for which it holds that $\left(c_{i},c_{i}^{\prime }\right)=\left(a_{i},b_{i}\right),\forall i\in \left[1,d\right]$. This implies that for P(M), it holds that d(P(M))=d and $\left(x_{i},y_{i}\right)=\left(a_{i},b_{i}\right),\forall i\in \left[1,d\right]$, and there does not exist a sequence $S=\left(\left(s_{1},s_{1}^{\prime }\right),\ldots ,\left(s_{n-1},s_{n-1}^{\prime }\right)\right)\in M\left(n,\Delta \right)$ for which it holds that $\left(s_{i},s_{i}^{\prime }\right)=\left(a_{i},b_{i}\right),\forall i\in \left[1,d\right]$ and $(\left(x_{1},y_{1}\right),\ldots ,\left(x_{n-1},y_{n-1}\right))≺S≺M$. This implies that for each such sequence *S*, it holds that P(M)(1)⊕P(M)(2)⊕⋯⊕P(M)(d)≻S(1)⊕S(2)⊕⋯⊕S(d). This and the definition of *k* imply that P(M)(i)=M(i), ∀i∈[1,k−1]. Furthermore, by Lemma 2(iii) and the definition of *p*, it holds that P(M)(i)≻P(M)(i+1), ∀i∈[k,k+p−1], and there does not exist a sequence $S=\left(\left(s_{1},s_{1}^{\prime }\right),\ldots ,\left(s_{n-1},s_{n-1}^{\prime }\right)\right)\in M\left(n,\Delta \right)$ such that $\left(s_{i},s_{i}^{\prime }\right)=\left(a_{i},b_{i}\right),\forall i\in \left[1,d\right]$ and S(i)≻S(i+1), ∀i∈[k,k+p−1] for which it holds that P(M)(k)⊕P(M)(k+1)⊕⋯⊕P(M)(K+1)≺S(k)⊕S(k+1)⊕⋯⊕S(k+p+1)≺M(k)⊕M(k+1)⊕⋯⊕M(k+p+1). Since $M\left(k\right)\neq S\left(a_{k},b_{k}\right)$, therefore P(M)(k) is a lexicographically minimum sequence in $M(a_{k},b_{k})$ for which it holds that P(M)(k)≺M(k). This implies that P(M)(k+i)=P(M(k)),∀i∈[0,p]. Further, by the minimality of P(M), P$\left(M\right)\left(i\right)=L\left(a_{i},b_{i}\right)$, ∀i∈[k+p+1,d].

Finally, one can easily verify that the sequence $\left(\left(x_{1},y_{1}\right),\ldots ,\left(x_{n-1},y_{n-1}\right)\right)$ obtained in each of the above cases satisfies Lemma 2(i)–(iii) by construction, and hence, $\left(\left(x_{1},y_{1}\right),\ldots ,\left(x_{n-1},y_{n-1}\right)\right)$ is an element of M(n,Δ) that is P(M), which completes the proof. □

**Lemma** **4.** *Let n≥2 and Δ≥0 be two integers and M be a sequence in M(n,Δ). Then, the predecessor* P*(M), if it exits, can be computed in O(n) time and O(n) space.*

The proof of Lemma 4 follows from Algorithm 1 and Lemma 5.

We next present Algorithm 1 to compute the predecessor based on Theorem 2. In this algorithm, for a sequence M∈M(n,Δ) with root-degree *d* and integer i∈[1,d], the variable M[i] stores the *i*-th root-subsequence M(i) of *M*, and the variable P[M] stores the predecessor, if it exists, of *M*.
**Algorithm 1** Computing the predecessor of an admissible sequence.**Input:** Two integers n≥2 and Δ≥0 and an (n,Δ)-admissible sequence $M=(\left(a_{1},b_{1}\right),\ldots ,\left(a_{n-1},b_{n-1}\right))$.**Output:** The predecessor *M* if it exists; The predecessor of *M* does not exist otherwise.1:d:= The root-degree of *M*;2:**if** If $a_{i}=1$ and $b_{i}=0$, ∀i∈[1,d]
**then**3: Output The predecessor of *M* does not exist /* Theorem 2(a) */4:**else**/* The predecessor of *M* exists by Theorem 2*/5: P$\left[M\right]:=(\left(x_{1},y_{1}\right),\ldots ,\left(x_{n-1},y_{n-1}\right))$6:  **if**
$a_{i}\neq 1$ for some i∈[1,d], and $b_{j}=0$ and $M\left[j\right]=S\left(a_{j},b_{j}\right)$, ∀j∈[1,d]
**then**   /* Theorem 2(b) */7:  $k:=\max\{i|a_{i}\neq 1\}$; $h:=k-1+\lceil \left(a_{k}+d-k\right)/\left(a_{k}-1\right)\rceil$; /* The root-degree   d(P(M)) of P(M) */8:  $y_{1}:=\Delta$; $y_{i}:=0,\forall i\in \left[2,h\right]$; $x_{i}:=a_{i},\forall i\in \left[1,k-1\right]$; $x_{i}:=a_{k}-1,\forall i\in \left[k,h-1\right]$;   $x_{h}:=a_{k}+d-k-\left\lfloor \left(a_{k}+d-k\right)/\left(a_{k}-1\right)\right\rfloor \left(a_{k}-1\right)$; P$\left[M\right]\left[i\right]=L\left(x_{i},y_{i}\right)$, ∀i∈[1,h].9: **else if**
$b_{i}\neq 0$ for some i∈[1,d] and $M\left[j\right]=S\left(a_{j},b_{j}\right)$, ∀j∈[1,d]
**then**   /* Theorem 2(c) */10:  $k:=\max\{i|b_{i}\neq 0\}$; $p:=\max\{i\leq d|a_{k}=a_{k+i}\}$; q:=p+1 if $b_{k}=1$;   $q:=\lfloor \left(\Delta -\sum_{1\leq i\leq k-1}b_{i}\right)/\left(b_{k}-1\right)\rfloor$ if $b_{k}\geq 2$; t:=min{q,p+1};11:   $x_{i}:=a_{i},\forall i\in \left[1,d\right];$$y_{i}:=b_{i},\forall i\in \left[1,k-1\right]$; $y_{k}:=b_{k}-1;$12:  **if**
k≠d
**then**13:   $y_{k+i}:=b_{k}-1,\forall i\in \left[1,t-1\right]$; $y_{k+t}:=\left(\Delta -\sum_{1\leq i\leq k-1}b_{i}\right)-t\left(b_{k}-1\right)$;   $y_{i}=0,\forall i\in \left[k+t+1,d\right]$14:  **end if**;15:  P$\left[M\right]\left[i\right]:=L\left(x_{i},y_{i}\right)$, ∀i∈[1,d]16: **else**/* If $a_{i}\neq 1,b_{j}\neq 0$ and $M\left[\ell \right]\neq S\left(a_{\ell },b_{\ell }\right)$, for some i,j,ℓ∈[1,d] */   /* Theorem 2(d) */17:  $k:=\max\{i|M\left[i\right]\neq S\left(a_{i},b_{i}\right)\}$;18:  $p:=\max\{i\leq d|a_{k}=a_{k+i}\;\mathrm{and}\;b_{k}=b_{k+1}\}$;   $\left(x_{i},y_{i}\right):=\left(a_{i},b_{i}\right),\forall i\in \left[1,d\right]$; P[M][i]:=M[i], ∀i∈[1,k−1];19:  P[M][k]:= Algorithm 1$\left(a_{k},b_{k},M\left[k\right]\right)$;20:  P[M][k+i]:=P[M][k],∀i∈[1,p]; P$\left[M\right]\left[i\right]:=L\left(a_{i},b_{i}\right)$, ∀i∈[k+p+1,d]21: **end if**22: Output P[M] as the predecessor of *M*;23:**end if**.

**Lemma** **5.** *For two integers n≥2 and Δ≥0 and an (n,Δ)-admissible sequence M, Algorithm 1 outputs the predecessor* P*(M), if it exits, in O(n) time and O(n) space.*

**Proof.**  Correctness: The correctness of Algorithm 1 immediately follows from Theorem 2.

Complexity analysis: By the definition of the root-degree, we can compute *d* at Line 3 in O(n) time.

We can test if $a_{i}\neq 1$ and $b_{j}\neq 0$ hold for some i,j∈[1,d] in O(n) time. Similarly, we can test if $M\left(j\right)=S\left(a_{j},b_{j}\right)$, for some j∈[1,d] in O(n) time, since the length of M(j) is $a_{j}-1$, and $\sum_{1\leq i\leq d}a_{i}=n-1$. Hence, we can test the conditions at Lines 2, 6, 9, and 16 in O(n+n)=O(n) time. This implies that we can check if the predecessor of *M* exists in O(n) time. We next discuss the time complexity of computing the predecessor in each of the cases at Lines 6, 9, and 16.

When the conditions at Line 6 hold, then *k* and *h* can be computed in O(n) time, since k≤d and h≤d+1. This implies that $\left(\left(x_{1},y_{1}\right),\ldots ,\left(x_{h},y_{h}\right)\right)$ can be computed in O(n) time. Furthermore, we can compute L$\left(x_{i},y_{i}\right),\forall i\in \left[1,h\right]$ in $O(x_{i})$ time by Lemma 3(i). Recall that for P[M], it holds that $\sum_{1\leq i\leq d}x_{i}=n-1$. Thus, P[M] can be computed in O(n) time.

When the conditions at Line 9 hold, then *k*, p,q, and *t* can be computed in O(n) time by there definitions. Thus, $\left(\left(x_{1},y_{1}\right),\ldots ,\left(x_{d},y_{d}\right)\right)$ can be computed from Line 11 to Line 14 in O(n) time. Furthermore, L$\left(x_{i},y_{i}\right),\forall i\in \left[1,d\right]$ can be computed at Line 15 in O(n) time, as discussed above. This implies that P[M] can be computed in O(n) time in this case.

Finally, when the conditions at Line 16 hold, then once again, we can compute *k* in O(n) time, since $\sum_{1\leq i\leq d}a_{i}=n-1$. Further, *p* can be computed in O(n) time by the definition of *p*. However, P[M][k] can be obtained by recursively running Algorithm 1 on $a_{k},b_{k}$ and M[k]. Note that this operation is repeated at most the length of the sequence P[M[k]], which is $a_{k}-1$ in this case, and hence, P[M][k] can be computed in O(n) time. Once again, the computation at Line 20 can be done in O(n) time since it holds that $\sum_{1\leq i\leq d}a_{i}=n-1$. Hence, P[M] can be computed in O(n) time.

Note that the O(n) space is sufficient to store P[M], if it exists, which completes the proof. □

Note that we can generate all rooted graphs in H(n,Δ) by generating their canonical representation by repeatedly using Algorithm 1 starting from the lexicographically maximum sequence L(n,Δ) in M(n,Δ) in O(n) time per graph and O(n) space in total.

**Theorem** **3.** *Let n≥2 and Δ≥0 be two integers. Then, all mutually non-isomorphic graphs with n vertices, Δ self-loops, and a tree skeleton can be generated in O(n) time per graph and O(n) space in total.*

**Proof.**  A tree can be viewed as a rooted tree by considering its centroid as the root [24]. We know that when *n* is odd, then there are only trees with unicentroids; however, when *n* is even, then there are trees with unicentroids.

By the definition of a unicentroid, all mutually non-isomorphic graphs with *n* vertices, Δ self-loops, and a tree skeleton with a unicentroid can be enumerated by generating all graphs *H* in H(n,Δ) such that each root subgraph of *H* has at most ⌊(n−1)/2⌋ number of vertices, i.e., by generating all sequences $M=\left(\left(a_{1},b_{1}\right),\ldots ,\left(a_{n-1},b_{n-1}\right)\right)\in M\left(n,\Delta \right)$ such that $a_{i}\in \left[1,\left\lfloor \left(n-1\right)/2\right\rfloor \right]$, ∀i∈[1,d(P(M))]. Let *S* denote the sequence that is lexicographically maximum among all those sequences in M(n,Δ) that represent a tree with a unicentroid. When *n* is even, then it holds that S=((⌊(n−1)/2⌋,Δ),(⌊(n−1)/2⌋,0),(1,0))⊕L(⌊(n−1)/2⌋,Δ)⊕L(⌊(n−1)/2⌋,0)⊕L(1,0). Recall that L(1,0) is an empty sequence, and therefore, we have S=((⌊(n−1)/2⌋,Δ),(⌊(n−1)/2⌋,0),(1,0))⊕L(⌊(n−1)/2⌋,Δ)⊕L(⌊(n−1)/2⌋,0). However, when *n* is odd, then it holds that S=((⌊(n−1)/2⌋,Δ),(⌊(n−1)/2⌋,0))⊕L(⌊(n−1)/2⌋,Δ)⊕L(⌊(n−1)/2⌋,0). Hence, we can generate all sequences in M(n,Δ) that represent a graph H∈H(n,Δ) such that the skeleton of *H* has a unicentroid by repeatedly using Theorem 2 starting from the sequence *S*. This implies that we can generate all such sequences in O(n) time per sequence and O(n) space by using Algorithm 1.

When *n* is even, then all mutually non-isomorphic graphs with *n* vertices, Δ self-loops, and a tree skeleton with a bicentroid can be enumerated by generating all sequences $M=\left(\left(a_{1},b_{1}\right),\ldots ,\left(a_{n-1},b_{n-1}\right)\right)\in M\left(n,\Delta \right)$ such that the root-degree d(M)=2, $a_{1}=a_{2}=n/2$ and $b_{1}+b_{2}=\Delta$ with $b_{1}\geq b_{2}$. In such a case, the sequences ((n/2,Δ),(n/2,0))⊕L(n/2,Δ)⊕L(n/2,0) and ((n/2,⌈Δ/2⌉),(n/2,⌊Δ/2⌋))⊕L(n/2,⌈Δ/2⌉)⊕L(n/2,⌊Δ/2⌋) are lexicographically maximum and minimum, respectively, among all those sequences in M(n,Δ) that represent a graph with a bicentroid. Let $M=\left(\left(a_{1},b_{1}\right),\ldots ,\left(a_{n-1},b_{n-1}\right)\right)\in M\left(n,\Delta \right)$ be a sequence that represents a graph H∈H(n,Δ) such that the skeleton of *H* has a bicentroid and M≠((n/2,⌈Δ/2⌉),(n/2,⌊Δ/2⌋))⊕L(n/2,⌈Δ/2⌉)⊕L(n/2,⌊Δ/2⌋). When $M\left(i\right)=S\left(a_{j},b_{j}\right)$, ∀i∈{1,2}, then it holds that P$\left(M\right)=\left(\left(a_{1},b_{1}-1\right),\left(a_{2},b_{2}+1\right)\right)⊕L\left(a_{1},b_{1}-1\right)⊕\left(a_{2},b_{2}+1\right)$. However, in the case otherwise, i.e., when $M\left(i\right)\neq S\left(a_{j},b_{j}\right)$, for some i∈{1,2}, then P(M) can be generated by using Theorem 2(d). Clearly, in both of these cases, P(M) can be generated in O(n) time and O(n) space. This eventually implies that all sequences that represent a graph in H(n,Δ) with a bicentroid can be generated in O(n) time per sequence and O(n) space by repeatedly computing the predecessor of sequences *M* as described above starting from ((n/2,Δ),(n/2,0))⊕L(n/2,Δ)⊕L(n/2,0) until M=((n/2,⌈Δ/2⌉),(n/2,⌊Δ/2⌋))⊕L(n/2,⌈Δ/2⌉)⊕L(n/2,⌊Δ/2⌋).

Hence, we can generate all non-isomorphic graphs with *n* vertices, Δ self-loops, and a tree skeleton with a unicentroid or bicentroid in O(n) time per graph and O(n) space in total, which completes that proof. □

## 4. Results and Discussion

We computed all graphs with *n* vertices, Δ self-loops, and a tree skeleton for different values of *n* and Δ to test the efficiency of our algorithm, and the results are listed in Table 1. These experiments were performed on a PC with an Intel Core i7-500 processor, running at 2.70 GHz, 16 GB of memory, and Windows 10. From the experimental results, it is evident that the proposed method is computationally efficient.

**An application to the generation of polymer topologies with a tree skeleton**: Observe that a graph with a tree skeleton, Δ self-loops, and no multiple edges has cycle rank Δ. Therefore, the class of such graphs contains all polymer topologies of cycle rank Δ with a tree skeleton. However, it is a natural question to search for a relationship between the number *n* of vertices and the number Δ of self-loops such that there exists a polymer topology with a tree skeleton, *n* vertices, and cycle rank Δ. Clearly, for n=1 and Δ≥2, there exists exactly one polymer topology with a tree skeleton, *n* vertices, and Δ self-loops. Let P(n,Δ) denote a maximal set of mutually non-isomorphic polymer topologies with a tree skeleton, *n* vertices, Δ self-loops, and no multiple edges. Azam et al. [21] proved the following necessary condition on Δ to have a polymer topology with a tree skeleton and *n* vertices.

**Lemma** **6** ([21])**.** *If n≥1 and $\Delta \geq \left\lceil \frac{n}{2}\right\rceil +1$, then it holds that P(n,Δ)≠∅.*

Let G(n,Δ) denote a maximal set of mutually non-isomorphic graphs with a tree skeleton, *n* vertices, and Δ self-loops. For an integer r≥1, let P(r) denote a maximal set of mutually non-isomorphic polymer topologies with a tree skeleton, *n* vertices, and *r* self-loops. By Lemma 6, it holds that:

(1) $$P\left(r\right)=\underset{n\in Z^{+}:\left\lceil \frac{n}{2}\right\rceil +1\leq r}{⋃}P\left(n,r\right)\subseteq \underset{n\in Z^{+}:\left\lceil \frac{n}{2}\right\rceil +1\leq r}{⋃}G\left(n,r\right)$$

Thus, by using Equation (1) and identifying the degree of each vertex in the graphs in G(n,r) from their canonical representations, we can compute all polymer topologies in P(r). We applied our method to generate all polymer topologies in P(r) for rank r=2,3,…,9, and the results are listed in Table 2.

## 5. Conclusions

We proposed an efficient method to enumerate all mutually non-isomorphic graphs with a tree skeleton, a given number of vertices, and the number of self-loops. The idea of this method is to generate rooted graphs with *n* vertices and Δ self-loops by generating their canonical representation. We defined the canonical representation of a rooted graphs *H* with *n* vertices and Δ self-loops based on the ordered graphs of *H*. The proposed method generates all graphs with a tree skeleton, *n* vertices, and Δ self-loops in O(n) time per tree and O(n) space in total. As an application, we can generate all polymer topologies with a tree skeleton, self-loops, no multiple edges, and a given cycle rank.

An interesting future research direction is to design a method that can directly count and enumerate all mutually non-isomorphic polymer topologies with a given cycle rank.

## Figures and Tables

**Table 1:** Experimental results of the enumeration method.

(n,Δ)	# of Generated Graphs	Time (s)
(5, 9)	856	0.278
(5, 10)	1186	0.213
(5, 30)	50,596	4.354
(6, 9)	4270	0.992
(6, 10)	6333	1.571
(6, 30)	619,431	141.334
(7, 9)	20,548	5.084
(7, 10)	32,337	7.047
(8, 9)	95,357	17.444
(8, 10)	159,058	31.755
(9, 9)	429,496	88.899
(9, 10)	756,045	185.823
(10, 9)	1,882,764	528.286
(10, 10)	3,488,567	914.806
(17, 2)	25,939,679	3911.33
(18, 0)	123,867	34.189
(20, 0)	823,065	334.357
(22, 0)	5,623,756	1807.53
(24, 0)	39,299,897	8042.88

**Table 2:** The number of polymer topologies with a tree skeleton, *r* self-loops, and no multiple edges.

	Rank *r*							
*n*	2	3	4	5	6	7	8	9
1	1	1	1	1	1	1	1	1
2	1	1	2	2	3	3	4	4
3	–	1	2	4	6	9	12	16
4	–	1	3	6	13	21	35	51
5	–	–	2	7	18	40	77	136
6	–	–	1	6	23	61	147	300
7	–	–	–	3	20	76	223	559
8	–	–	–	1	14	74	288	868
9	–	–	–	–	5	54	291	1128
10	–	–	–	–	2	29	241	1212
11	–	–	–	–	–	10	145	1057
12	–	–	–	–	–	2	68	733
13	–	–	–	–	–	–	19	390
14	–	–	–	–	–	–	4	151
15	–	–	–	–	–	–	–	38
16	–	–	–	–	–	–	–	6
Total	2	4	11	30	105	308	1555	6650
